# Resignations and Appointments in Medical Schools

**Published:** 1858-05

**Authors:** 


Resignations and Appointments in Medical Schools.—We have learned with profound regret that Dr. Miller, who had so long held the chair of Obstetric Medicine in the University of Louisville, has resigned his office. The announcement of his retirement will be received by the medical profession throughout the country with sorrow, especially in the Southwest, where he is so well known and so justly appreciated for his many excellent qualities as a man, a practitioner, and a teacher. Connected with the Institution from its earliest inception, no one who has ever been associated with it has worked harder, more faithfully, or more successfully for the promotion of its prosperity. We sincerely hope that the Trustees and Alumni of the School will make an effort to recall him to a post which he has filled with so much ability, and in which no one, in all the Southwest, can replace him. Experience comes not in a day, nor is reputation attained without toil.

Professor R. K. Thomas, in consequence of ill-health, has resigned the chair of Obstetrics in the University of Maryland, which he held for some years. His successor is Dr. G. W. Miltenberger, formerly Professor of Materia Medica and Therapeutics in the same institution. To the chair thus vacated Dr. Charles Frick has been appointed, and we are sure no better selection could possibly have been made. We know Dr. Frick well, and, independently of his many excellent social qualities, there is no man of his age in the country who is better qualified for such a chair.

Dr. Joseph Jones, recently Professor of Chemistry in the University of Georgia, has been appointed to the chair of Chemistry and Pharmacy in the Medical College at Augusta, rendered vacant by the retirement of Professor Means. This gentleman, although but a recent graduate of medicine, is already favorably known to the profession by his excellent contributions to chemical and physiological science. These contributions, founded as they are upon laborious personal observation, have achieved for Dr. Jones a high position in the profession, and augur for him a most brilliant career. We congratulate the medical faculty at Augusta upon the acquisition to their corps of so devoted a student and able investigator.

